# Rostro-Caudal and Caudo-Rostral Migrations in the Telencephalon: Going Forward or Backward?

**DOI:** 10.3389/fnins.2017.00692

**Published:** 2017-12-21

**Authors:** Nuria Ruiz-Reig, Michèle Studer

**Affiliations:** Université Côte d'Azur, CNRS, Inserm, iBV, Nice, France

**Keywords:** cortical interneurons, telencephalon, amygdala, olfactory bulb, transcription factors, neuronal migration, subpallium, pallium

## Abstract

The generation and differentiation of an appropriate number of neurons, as well as its distribution in different parts of the brain, is crucial for the proper establishment, maintenance and plasticity of neural circuitries. Newborn neurons travel along the brain in a process known as neuronal migration, to finalize their correct position in the nervous system. Defects in neuronal migration produce abnormalities in the brain that can generate neurodevelopmental pathologies, such as autism, schizophrenia and intellectual disability. In this review, we present an overview of the developmental origin of the different telencephalic subdivisions and a description of migratory pathways taken by distinct neural populations traveling long distances before reaching their target position in the brain. In addition, we discuss some of the molecules implicated in the guidance of these migratory paths and transcription factors that contribute to the correct migration and integration of these neurons.

## Introduction

In the nervous system, neurons are generated from progenitors distributed in the ventricular and subventricular zones (VZ and SVZ), a territory located in the apical part of the neural tube next to the ventricles, and representing active proliferative germinal zones. During embryonic development, these progenitors divide symmetrically and asymmetrically to produce more progenitors and neurons, increasing in this way the pool of cells in the nervous system. Early postmitotic neurons migrate subsequently to their correct positions to acquire a proper function and integrate into a distinct brain circuitry. Neurons make use of different mechanisms to travel along the brain surface and within the nervous tissue: radial and tangential migration. In the radial migration, neurons follow the radial glia moving from the VZ to the pia perpendicular to the apical surface, whereas in the tangential migration, newborn neurons move perpendicular to the radial glia and therefore parallel to the ventricular surface (revised by Valiente and Marín, 2010; Hatanaka et al., 2016).

We will focus mainly on the telencephalon, which is subdivided into pallial (dorsal telencephalon) and subpallial (ventral telencephalon) territories. Neurons generated in these different regions migrate radially and tangentially to distribute along the whole telencephalic vesicles. While the pallium produces glutamatergic excitatory neurons, the subpallium is the source of mainly GABAergic inhibitory neurons, but both populations will colonize pallial and subpallial territories by radial and tangential migration (de Carlos et al., 1996; Anderson et al., 1997; Marín and Rubenstein, 2001). In most cases, neurons are generated close to their final destination and thus migrate only short distances. However, some neurons need to migrate long distances before reaching their final position in the brain, adding thus a new dimension along the rostro-caudal axis. This new aspect includes a simultaneous combination of radial and tangential migration. Since several of these migratory paths occur at the same time, the telencephalon becomes a very busy territory with neurons moving in all kind of directions.

But how do cells know where to go and when to stop and ultimately settle down? There is still much work to do to elucidate the complete mechanisms that guide neurons along their migratory paths in the developing brain. When neurons are born, they will express a distinct cell-intrinsic genetic program that will instruct them about their identity but also about their final destinations; this molecular program includes a whole series of receptors, mainly expressed in structures resembling growth cones at the end of the leading process, that will help them to find their correct path (Nóbrega-Pereira and Marin, 2009). Neurons explore their territory by extending novel branches (Bellion et al., 2005) and continuously changing the angle of these branches (Martini et al., 2009). Chemomolecules in the migratory substrate are detected by specific receptors that will endorse neurons to retract and remodel their leading process (Polleux et al., 2002; Kalil and Dent, 2005; Martini et al., 2009). Once they find their correct environment, the nucleus translocates to the new branch and the trailing process retracts allowing thus proper cell movement (Morris et al., 1998). Most of the guidance cues include the same types of molecules implicated in other systems to guide axonal tracts, and mainly represent members of the semaphorins, netrins and slit families (Brose and Tessier-lavigne, 2000; Bagri and Tessier-lavigne, 2002; Marín et al., 2010).

In this review, we are going to focus on specific neural populations that migrate long distances along the rostro-caudal axis of the telencephalon. At this point, it becomes crucial to clarify what we mean by “rostro-caudal axis.” According to the prosomeric model, the neural tube is subdivided into antero-posterior (A-P) segments, or neuromers, established by genetic and morphological boundaries (Bulfone et al., 1993; Puelles et al., 2013). The secondary prosencephalon (that comprise the hypothalamus and the telencephalon) is located in the most rostral part of the neural tube. The telencephalon is a huge expansion of the alar plate of the secondary prosencephalon and the relations with the topological A-P axis are partially lost. We take into account that the most topological rostral edge of the telencephalon is aligned with the preoptic area, however, to simplify the terminology, we will use the anatomical A-P axis and locate the rostral/anterior part of the telencephalon in the olfactory bulbs, while the most caudal/posterior pole would correspond to the posterior amygdala and entorhinal cortex.

In summary, we will introduce the paths taken by GABAergic and glutamatergic neurons to migrate either to more rostral (going “forward”) or more caudal (going “backward”) territories of the telencephalon. Furthermore, we will discuss some of the chemoattractive and chemorepulsive signals required in guiding these populations along their long paths, as well as the transcription factors (TFs) expressed by these neurons implicated in distinct migratory steps.

## GABAergic neurons utilize different migratory routes before reaching their final targets

The subpallium is subdivided during development into ganglionic eminences, preoptic area (POA) and subpallial septum. The ganglionic eminences (GE) themselves can be subdivided into three major proliferative regions, called lateral (LGE), medial (MGE), and caudal ganglionic eminences (CGE). It is now well accepted that the different subdivisions of the ventral telencephalon are established according to discrete combinations of TFs they express during development (Puelles et al., 2000; Schuurmans and Guillemot, 2002; Flames et al., 2007). Each subdivision generates distinct GABAergic subpopulations assigned to different pallial structures, as well as to basal ganglia and amygdala of the ventral telencephalon. Once they have reached their destinations, GABAergic neurons can act as local circuit neurons (interneurons) or principal projection neurons. The best example of GABAergic migration during telencephalic development is represented by cortical interneurons (cINs), which are generated mainly in the MGE (60%) and CGE (30%), and to a minor degree in the POA (10%). Specific molecular and temporal codes determine the identity of cINs and their distribution in the cortex (reviewed in Sultan and Shi, 2017).

Interneurons generated in the subpallium migrate first tangentially to reach the cortex and then radially using a particular migratory behavior known as “random walk,” to finally settle in a specific cortical layer (Lopez-Bendito et al., 2008; Kelsom and Lu, 2013). However, no matter where they are generated, cINs are distributed in the whole cortex along all axes. This implies that they must migrate radially and tangentially, and along the rostral and caudal directions to colonize the entire cortical plate. Since several studies are mainly focused on the mechanisms guiding MGE-derived interneurons toward the cortex (revised in Marín, 2013), we will give more emphasis on what is known about the rostro-caudal migratory paths of the CGE, POA, and dLGE subpopulations. In the next paragraphs, we will describe these migratory routes and their transcriptional signature.

### From the CGE rostrally and caudally

**Figure 1:**
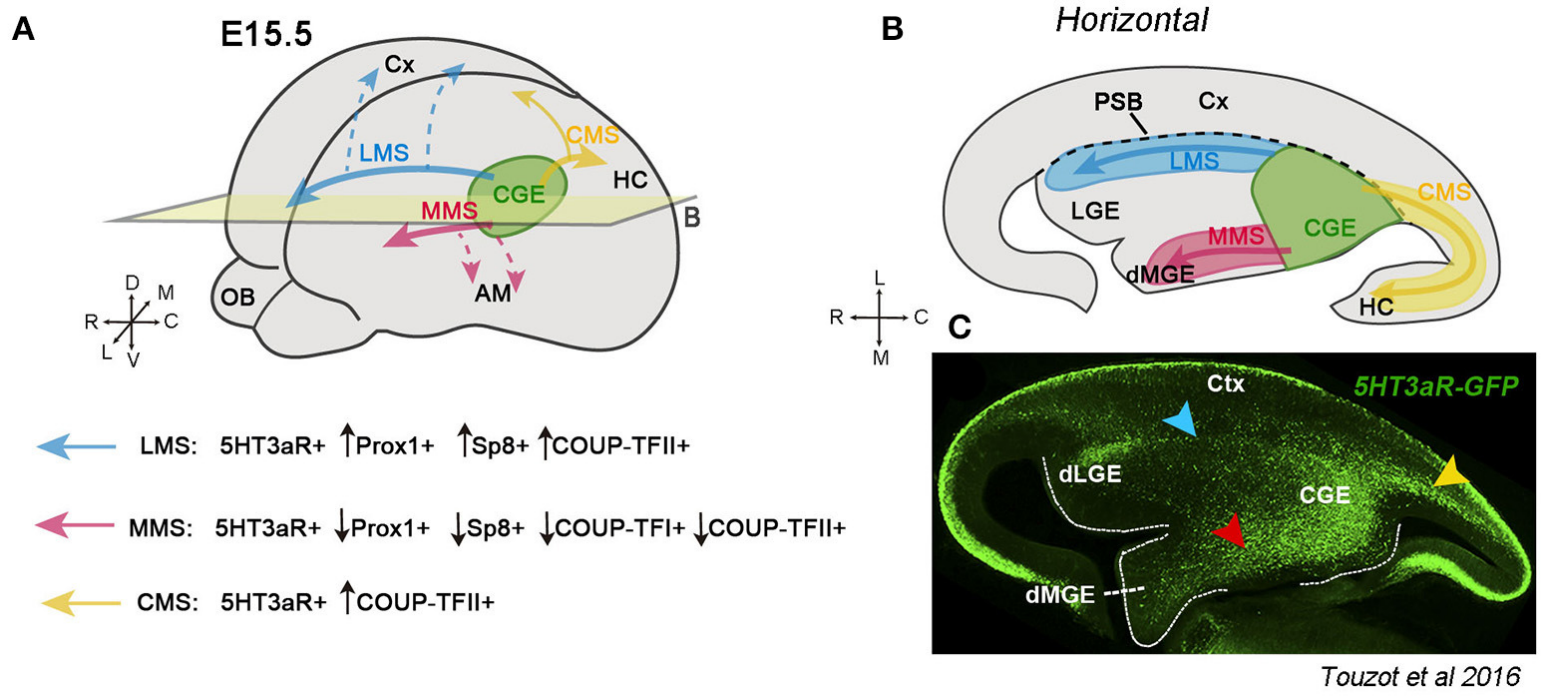
Migratory streams originating from the caudal ganglionic eminence (CGE) during mouse development. **(A)** Schematic representation of the telencephalic vesicles at embryonic (E) stage 15.5. The CGE (in green) generates GABAergic neurons that migrate laterally through the LMS (blue arrow), medially along the MMS (red arrow) or caudally *via* the CMS (yellow arrow). Neurons taking the LMS are positive for the serotonin receptor *5HT3aR* and a high percentage also express Prox1 and Sp8. The LMS helps CGE neurons to get distributed along the cortex (Cx) (dashed blue arrows). Neurons migrating through the MMS are *5HT3aR-GFP*+, but only a low percentage express Prox1, Sp8 or COUP-TFI and II. The most putative target of neurons migrating along the MMS is the amygdala (AM) (dashed red arrows). The CMS is composed of neurons expressing *5HT3aR* and a high percentage the COUP-TFII. CGE neurons migrate through the CMS toward the hippocampus (HC) and caudal cortex (yellow arrows) (Yozu et al., 2005; Kanatani et al., 2008, 2015). **(B)** Illustration of a horizontal section at the level of the plane indicated in **A** showing the different CGE-derived migratory streams. The black dashed line represents the boundary between the cortex and the subpallium (PSB). **(C)** Immunostaining of a horizontal section showing the distribution of *5HT3aR-GFP*+ cells in the CGE, the LMS (blue arrowhead), MMS (red arrowhead) and CMS (yellow arrowhead) during embryonic development. Image taken from Touzot et al. (2016).

The CGE is the caudal portion of the ganglionic eminences and mainly generates GABAergic interneurons characterized by the expression of TFs such as Prox1, Sp8, COUP-TFI (also called Nr2f1) and COUP-TFII (Nr2f2), in addition to the expression of the ionotropic serotonin receptor 3a (5HT3aR) (Tripodi et al., 2004; Yozu et al., 2005; Kanatani et al., 2008; Vucurovic et al., 2010; Lodato et al., 2011; Rudy et al., 2011; Ma et al., 2012; Cai et al., 2013; Rubin and Kessaris, 2013). CGE-derived neurons are distributed in the cerebral cortex, hippocampus, amygdala and striatum (Nery et al., 2002; Yozu et al., 2005; Lee et al., 2010; Miyoshi et al., 2010; Vucurovic et al., 2010; Chittajallu et al., 2013; Muñoz-Manchado et al., 2016; Torigoe et al., 2016; Touzot et al., 2016). To reach these multiple destinations, CGE interneurons take different migratory routes during mouse development. The Caudal Migratory Stream (CMS) was the first described migratory path for CGE neurons, as demonstrated by focal CGE electroporations in telencephalic whole-mount cultures in which a subset of CGE-derived neurons was shown to migrate caudally toward the hippocampus and caudal cortex (Yozu et al., 2005) (yellow arrows Figures 1A,B). Recently, two additional streams have been described for CGE neurons migrating rostrally (Touzot et al., 2016). Taking advance of the *5HT3aR* reporter mouse line in which the majority of CGE-derived neurons are labeled thanks to the fluorescent protein GFP, Touzot et al., showed that CGE-derived cells also migrate rostrally taking a lateral path through the pallial-subpallial boundary (PSB), and a medial path crossing the dorsal MGE (Figures 1A,B, blue and red arrows, respectively). These two new migratory routes have been named “Lateral and Medial Migratory Streams (LMS and MMS)” respectively, and GFP+ cells traveling within each stream express different proportions of CGE TFs (Figure 1). For example, neurons in the CMS express mainly COUP-TFI and COUP-TFII and to a minor degree Sp8 and Prox1 (Kanatani et al., 2008; Touzot et al., 2016). COUP-TFII plays a key role in the migration taken by cells within the CMS. Indeed, inhibition of COUP-TFII using *siRNA* prevents the neurons generated in the CGE to migrate caudally and, conversely, overexpression of COUP-TFII in MGE-derived cells and then transplantation of these cells into the CGE induces grafted cells to take a caudal path *via* the CMS (Kanatani et al., 2008). Thus, COUP-TFII is necessary and sufficient for driving CGE-derived cells toward caudal directions.

The LMS contains neurons expressing preferentially Sp8, Prox1, and COUP-TFII (Touzot et al., 2016). The LMS cross the PSB at the level of where dLGE interneurons are generated and destined to the olfactory bulbs and cortical white matter (Stenman et al., 2003; Inta et al., 2008; Frazer et al., 2017; blue arrows in Figures 1A,B). Albeit both populations share *5HT3aR-GFP* and Sp8 expression, CGE interneurons can be distinguished from dLGE-derived interneurons because they express Prox1 and COUP-TFII, but not Er81 and Meis2, which are instead markers of migrating olfactory bulb interneurons (Allen et al., 2007; Inta et al., 2008; Vucurovic et al., 2010; Chen R. et al., 2012; Ma et al., 2012; Cai et al., 2013; Rubin and Kessaris, 2013; Agoston et al., 2014; Touzot et al., 2016; Frazer et al., 2017). Moreover, homotopical CGE transplantations in organotypic cultures and fate map experiments using cell trackers support the lateral path taken by CGE neurons to migrate rostrally (Touzot et al., 2016). Neurons taking the LMS are very likely to migrate to the cortex since the same combination of TFs (Prox1, Sp8, and COUP-TFII) is also shared by neocortical CGE interneurons. Additionally, the LMS is well recognizable between E13.5 and E15.5 corresponding to the peak of CGE-cIN production (Touzot et al., 2016). Thus, the LMS could represent a crucial migratory path allowing CGE interneurons to disperse along the entire cerebral cortex (blue dashed arrows, Figure 1A).

Finally, the MMS contains *5HT3aR-GFP*+ neurons migrating rostrally and medially from the CGE to the dorsal MGE crossing the bed nucleus of the stria terminalis (BST) (red arrows in Figure 1). Regarding the transcriptional signature, MMS neurons express to a lesser extent COUP-TFII, Sp8 and Prox1 when compared to the CMS and LMS. The final destination of CGE neurons taking the MMS is very likely to be the amygdala complex (red dashed arrows, Figure 1A), as supported by the high percentage of *in utero* grafted CGE/GFP+ homochronic neurons in the adult amygdalar region, particularly in the basolateral complex and medial amygdala (Vucurovic et al., 2010; Touzot et al., 2016).

Besides regulating cell migration, COUP-TFII and its homolog COUP-TFI are also involved in the generation and final distribution of both CGE- and MGE-derived cINs. Conditional deletion of COUP-TFI in the SVZ ganglionic eminence leads to an over-proliferation of committed progenitors in both MGE and CGE. As a result, the LMS and MMS exhibit an increased number of *5HT3aR-GFP*+ neurons migrating through these paths. Interestingly, COUP-TFI not only regulates the generation of CGE neurons but also the ratio of different TFs that these neurons express within each migratory stream. Deletion of COUP-TFI increases the percentage of neurons expressing COUP-TFII in the MMS and Sp8 in the LMS, leading thus to altered specification of cIN subtypes, and in line with their role as cell fate determinant genes (Touzot et al., 2016). Indeed, COUP-TFI represses rostral MGE and promotes CGE identity, and in conjunction with COUP-TFII, both specify Somatostatin (SST) fate in the caudal MGE and repress Parvalbumin (PV) identity in the rostral MGE (Lodato et al., 2011; Touzot et al., 2016; Hu et al., 2017). As a result, COUP-TFI interneuron-specific mutants exhibit an imbalanced proportion of MGE- and CGE-derived cINs in the adult neocortex (Lodato et al., 2011). These data uncovered an unexpected complexity in the migratory paths and transcriptional control of MGE- and CGE-derived GABAergic cINs necessary to generate the huge diversity of mature interneuron subtypes in the brain.

The serotonin receptor 5HT3aR expressed in migrating CGE cINs plays instead a key role in controlling their migratory speed during invasion to the cortical plate (CP) and at late phases of migration. Loss of *5HT3aR* produces a defect in the integration of reelin (RLN)-expressing cells in the superficial layer 1 of the neocortex, and as a consequence, a laminar mis-positioning of RLN+ cINs derived from the CGE (Murthy et al., 2014). More than during early specification, Prox1 is mainly involved in the maturation of CGE cINs. During CGE cortical migration, Prox1 controls the integration of CGE-derived interneurons in superficial layers, as well as the morphology and properties of these cells (Miyoshi et al., 2015). All these studies show that cell fate determination and migratory decisions are strictly linked and tightly controlled during interneuron development. Thus, committed interneuron progenitors must express a distinct combination of transcriptional regulators that cell-intrinsically control their migratory choice and contribute to their distinct laminar position in the adult brain. We are just starting to untangle the complex molecular network regulating the different properties of GABAergic interneurons in the cerebral cortex.

### From the preoptic area (POA) to the medial amygdala and caudal cortex

**Figure 2:**
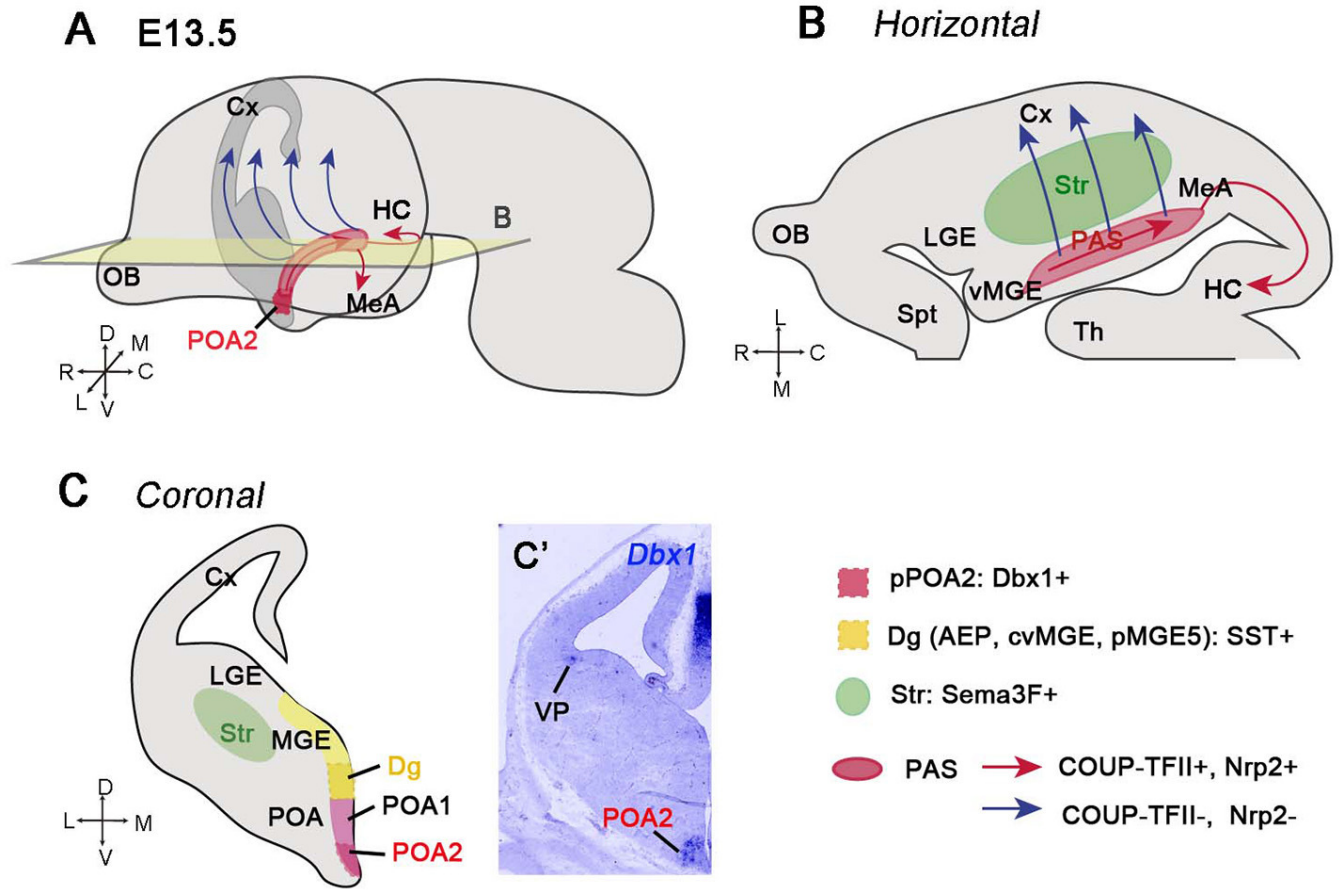
GABAergic neurons generated from the POA2 region migrate caudally through the posterior amygdala stream (PAS). **(A)** Illustration of a whole embryonic brain at E13.5 showing the migratory paths (red and blue arrows) taken by the GABAergic neurons generated from the POA2 region, represented in dark red color. **(B)** Schematic of a horizontal section at the level of the plane shown in **A**. Neurons generated from the POA2 region express COUP-TFII and Nrp2 and migrate toward the HC, MeA, and caudal Cx *via* the PAS and CMS (red arrows). The red area represents the PAS. Neurons that downregulate COUP-TFII and therefore Nrp2 (blue arrows) detach from PAS and migrate across the striatum (Str, green color area) toward the cerebral cortex. **(C)** Schematic of a coronal section showing the different areas from which newly-generated GABAergic neurons migrate caudally. The POA is subdivided into POA1 and POA2 subregions while the Dg (also called AEP, cvMGE or pMGE by different authors) corresponds to the ventral region of the MGE. The POA2 progenitor domain (pPOA2) contains Dbx1+ progenitors, which produce GABAergic neurons migrating through the PAS (Hirata et al., 2009; Kanatani et al., 2015). The POA1 mantle (POA1m) is Nkx5.1+ but fail to generate neurons migrating caudally (Gelman et al., 2009; Kanatani et al., 2015). The Dg generates somatostatin (SST)-expressing neurons probably by also taking a migratory stream toward the caudal telencephalon (Puelles et al., 2016b). **(C**′**)** *In situ* hibridization showing *Dbx1* expression in the POA2 region at E12.5.

The POA is a region situated in the ventral part of the telencephalon. Although some authors consider this region as part of the diencephalon (Kanatani et al., 2015), the POA is located at the border of the subpallium with the alar hypothalamus, and expresses the telencephalic marker Foxg1 (Tao and Lai, 1992; Hirata et al., 2009; Medina et al., 2011; Bupesh et al., 2011b; Ferran et al., 2015). The POA is subdivided into two different regions (POA1 and POA2) depending on the expression profile of different TFs (Flames et al., 2007; Figure 2). The POA2 domain is characterized by the expression of Dbx1 and generates GABAergic neurons destined to the cerebral cortex and amygdala (Gelman et al., 2009, 2011; Hirata et al., 2009; Kanatani et al., 2015; Figures 2A–C′ dark red region Figure 2C). These Dbx1-expressing derivatives from the POA2 region are cINs and projecting amygdalar neurons. In the cerebral cortex, POA-Dbx1 derivatives mature into PV+ and SST+ interneurons of lower cortical layers (Gelman et al., 2011), whereas in the amygdala, they are situated mainly in the medial amygdala (MeA), where the majority express nNOS as a marker of medial nuclei projection neurons (Hirata et al., 2009; Carney et al., 2010). The way these cells reach caudal regions of the telencephalon or the cerebral cortex was nicely illustrated by Hirata et al. (2009) and Kanatani et al. (2015). Using the *Dbx1* reporter mouse line, Hirata et al. showed that GABAergic neurons generated from Dbx1+ progenitors in the POA2 region migrate towards the caudal part of the telencephalon. This migratory stream was called Preoptic Amygdala Stream (PAS) (Figure 2B). Focal electroporation in the POA2 region confirmed the existence of this path (Kanatani et al., 2015), even if the authors included it as part of the CMS. Electroporated cells could reach the MeA (at the level of the CGE), while other cells continued to migrate farther to the caudal cortex and hippocampus or laterally toward the neocortex. Neurons generated in the POA2 express the MGE marker Lhx6, but also COUP-TFII, which controls the expression of the Semaphorin Sema3F receptor Neuropilin 2 (Nrp2) and allows cells to migrate through the PAS-CMS toward the medial amygdala and caudal cortex (red arrows Figures 2B,C). The population that downregulates COUP-TFII and thus Nrp2 expression, can cross the Sema3F-expressing striatum and migrate laterally to the cerebral cortex (Marin et al., 2001; Lin et al., 2010; Tang et al., 2012; Kanatani et al., 2015) (blue arrows in Figures 2A,B). Moreover, knocking down *COUP-TFII* or *Nrp2* by *shRNA* resulted in an increased number of cells reaching the cortex rostrally, and conversely, overexpressing these two factors produced an accumulation of neurons in the medial amygdala. This molecular switch between COUP-TFII and Nrp2 allows thus neurons generated in the POA2 to be differently distributed. The CGE interneurons destined to the caudal cortex and hippocampus take the same path as POA2 GABAergic neurons through the CMS (Yozu et al., 2005). The role of COUP-TFII thus seems to be similar in both populations (Kanatani et al., 2008, 2015) and allows interneurons *via* Nrp2 to properly respond to their environment.

### The controversial origin of somatostatin (SST) interneurons

There are however also other GABAergic neuron populations taking the same caudal migratory path through the PAS-CMS. This is the case of SST+ neurons generated in the diagonal area (Dg) of the telencephalon. The Dg, previously called anterior endopeduncular area (AEP), caudal or caudo-ventral MGE (cMGE, cvMGE), is the region situated between the POA and the pallidum in the pMGE5 subdomain, according to the characterization of Flames et al. (2007; dark yellow region in Figure 2C). The Dg is the most likely origin of the SST+ neurons in the telencephalon (Garcia-Lopez et al., 2008; Real et al., 2009; Bupesh et al., 2011a; Puelles et al., 2016b; Hu et al., 2017), although this population was previously proposed to be generated in the dorsal part of the MGE (Xu et al., 2004; Butt et al., 2005; Flames et al., 2007; Fogarty et al., 2007; Ghanem et al., 2007; Wonders et al., 2008; Sousa et al., 2009). The final targets of SST+ neurons include pallial and subpallial structures, such as the neocortex, claustrum, pallidum, and striatum, as well as the caudal telencephalon, such as the entorhinal cortex, medial and cortical amygdala and hippocampus. Based on the SST expression profile at early stages of mouse development, it was suggested that SST+ neurons destined to the posterior telencephalon migrate caudally through the amygdala along a ventral stream named by L. Puelles, Subventricular Subpallial Migratory stream (SvSpM) (Puelles et al., 2016b). This SST+ migratory path is probably the same described by Medina and colleagues in horizontal organotypic cultures (Bupesh et al., 2011a,b). In these experiments, they showed that cells labeled in the Dg (or cvMGE) with a cell tracker are calbindin+ neurons migrating caudally to the posterior medial amygdala. Although no co-labeling with SST was shown, this stream of cells most probably corresponds to the SST+ neurons previously shown in horizontal sections (Bupesh et al., 2011a). In addition, the same authors noticed that some of the cells generated in the Dg leave the main stream and start to migrate laterally toward the striatum, dorsal pallidum, globus pallidum and piriform cortex; however, due to the technical limitations of horizontal sections, they could not conclude whether these cells can also reach the cortex.

These studies describing neurons originating from a ventral MGE region and traveling caudally make us wonder whether they all refer to the same population or, on the contrary, they represent different GABAergic subpopulations undertaking the same path, before reaching the posterior telencephalon. In the neocortex, some of the cINs generated from Dbx1-expressing progenitors are co-labeled with SST (Gelman et al., 2011), suggesting a partial origin of this population from the POA. However, in the case of the postero-medial amygdala (MeP), Dbx1-derivatives and SST+ neurons represent most probably two distinct populations distributed in different subnuclei. Differently, nNOS projecting neurons generated in the POA2 are localized mainly in the postero-ventral medial amygdala (MePV), whereas SST+ neurons are localized primordially in the dorsal part (MePD) (Hirata et al., 2009; Carney et al., 2010; Puelles et al., 2016a,b). It is anyway remarkable that both populations take the same migratory pathway to arrive at the posterior amygdala, even if it is still unclear whether they use the same cues and strategy to reach their final targets.

### From the dLGE-SVZ to the olfactory bulb (OB)

**Figure 3:**
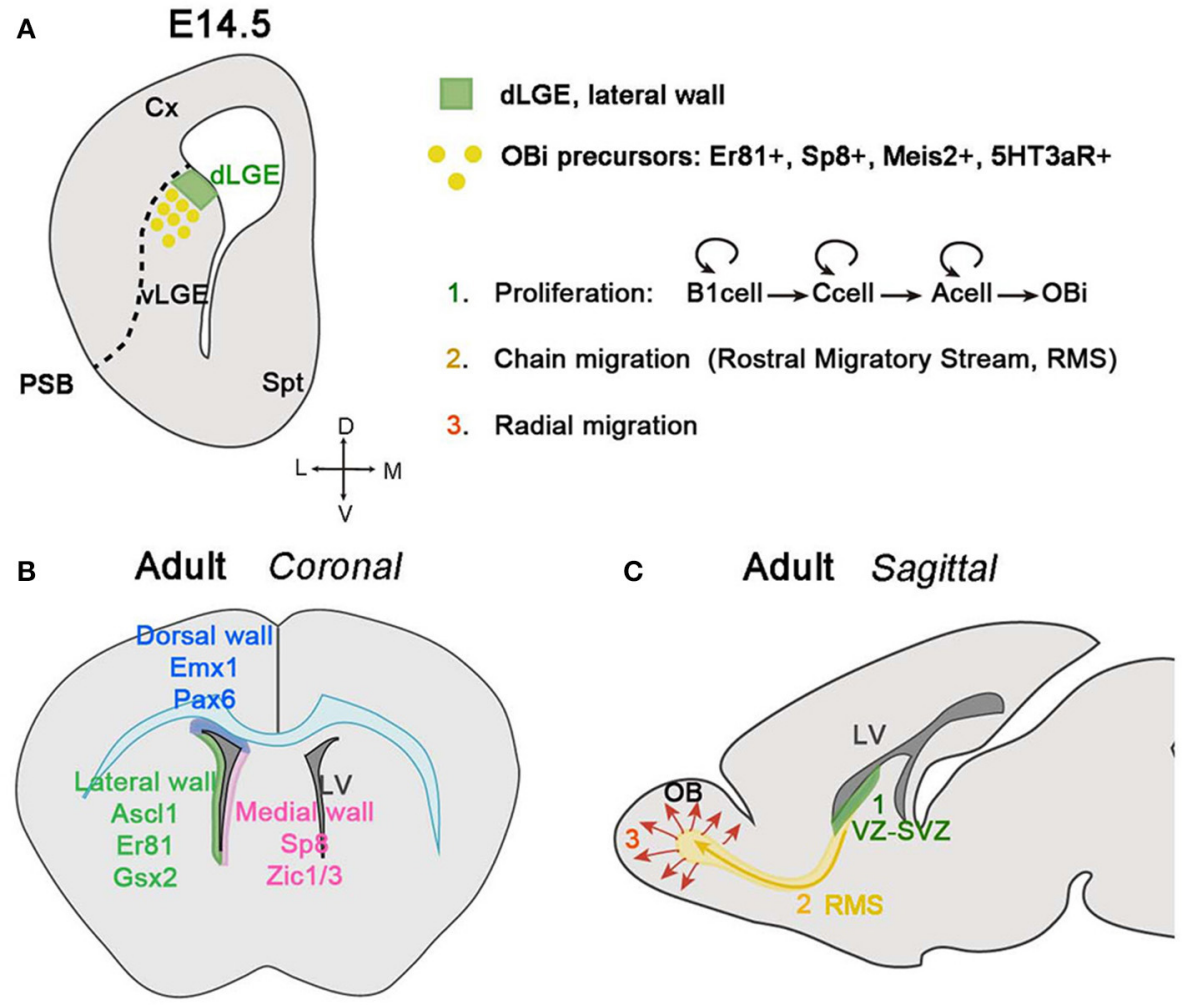
Olfactory bulb interneuron (OBi) generation and migration during development and adulthood. **(A)** Schematic of a coronal section at E14.5 showing the origin of OBi in the dLGE. The VZ of the dLGE (green area), situated at the border of the PSB, generates OBi precursors expressing Er81, Sp8, Meis2 and the receptor 5HT3aR. **(B)** Schematic of a coronal section showing the neurogenic niche of OBi during adult stages. The VZ-SVZ in the dorsal wall facing the pallium and corpus callosum (represented in blue color) generates OBi from Emx1+ and Pax6+ progenitors (Hack et al., 2005; Kohwi et al., 2005, 2007; Seri et al., 2006; Willaime-Morawek et al., 2006; Merkle et al., 2007; Ventura and Goldman, 2007; Young et al., 2007). The VZ-SVZ in the medial wall facing the septum (represented in pink color) express Sp8 and Zic1/3 (Morshead et al., 1994; Waclaw et al., 2006; Inoue et al., 2007; Merkle et al., 2007). The VZ-SVZ in the lateral wall facing the striatum (represented in green color) express Ascl1 (also known as Mash1), Er81 and Gsx2 (Casarosa et al., 1999; Stenman et al., 2003; Parras et al., 2004; Allen et al., 2007; Berninger et al., 2007; Young et al., 2007; Waclaw et al., 2009). **(C)** Schematic of a sagittal section of an adult mouse brain showing the different steps in the generation, migration, and integration of OBi. Proliferation (in green): stem cells located in the VZ-SVZ of the lateral ventricles (B1 cells) can divide asymmetrically for self-renewal and produce transit-amplifying precursors (type C cells). Finally, type C cells give rise to neuroblasts (type A cells) that can also undergo divisions while they are migrating through the RMS to finally differentiate into OBi once they reach the OB. Chain migration (in yellow): neuroblasts migrate forming chains among them and with the help of glial tubes, along the RMS before reaching the OB. Radial migration (in red): once OBi neuroblasts reach the OB, they detach from the RMS and migrate radially to integrate into the different OB layers.

Olfactory bulb interneurons (OBi) are a peculiar subset of local connection neurons that modulate the olfactory circuitry, which depends on activity from late postnatal to adult stages (revised in Lledo and Valley, 2016). Since this population represents one of the two neurogenic niches generated during adult stages in mammals, several studies have focused on understanding how the different steps of adult neurogenesis are molecularly controlled, and how these stem cells migrate and integrate into the OB. Interestingly, OBi are not only composed of GABAergic neurons, but also of dopaminergic tyrosine hydroxylase (TH) cells and of a small subpopulation of glutamatergic juxtaglomerular interneurons (Halász et al., 1982; Baker et al., 1983; McLean and Shipley, 1988; Brill et al., 2008). In general, OBi can be classified into periglomerular (PGCs) and granule cells (GCs), each group receiving distinct inputs and controlling the OB circuitry in different ways (revised in Kosaka and Kosaka, 2016; Lledo and Valley, 2016). PGCs surround the glomeruli and are subdivided into three different non-overlapping subtypes depending on the expression of calretinin, calbindin, and TH (Kosaka and Kosaka, 2005; De Marchis et al., 2007; Kohwi et al., 2007). GCs instead are primarily calretinin-expressing cells located in the mitral and granular cell layers (MCL and GCL, respectively). Although several studies have mainly focused on OBi neurogenesis during adult life, it is noteworthy to point out that OBi are also generated during development from a restricted dorsal portion of the LGE (dLGE) located at the PSB (Bulfone et al., 1998; Goldman and Luskin, 1998; Wichterle et al., 1999; Stenman et al., 2003), and from the prospective OB (pOB) (Vergaño-Vera et al., 2006). As previously mentioned, OBi precursors of the dLGE express Er81, Sp8, Meis2 and the serotonin receptor *5HT3aR* (Figure 3A), in contrast to progenitors located in the ventral LGE (vLGE) and expressing the TF Islet 1 (ISL1). These cells will give rise to striatal neurons instead of interneurons (Yun et al., 2001, 2003; Stenman et al., 2003; Allen et al., 2007; Inta et al., 2008; Waclaw et al., 2009; Chen Y. et al., 2012; Agoston et al., 2014; Touzot et al., 2016).

The first OBi are generated around E12.5 and reach the OB around E14.5 in the mouse (Kaplan et al., 1985; Wichterle et al., 2001; Pencea and Luskin, 2003; Stenman et al., 2003; Yoshihara et al., 2005; Tucker et al., 2006; Batista-Brito et al., 2008). However, while the peak of production of OBi is perinatal, during the first week of postnatal life in rodents, neurogenesis decline gradually through adulthood (Hinds, 1968; Lledo et al., 2008; Batista-Brito and Fishell, 2009; Díaz-Guerra et al., 2013). The inducible *Dlx1/2*-*Cre* mouse combined with a reporter line showed that the first generated OBi are dopaminergic TH- and calbindin-expressing cells, both contributing to the PGC population (Batista-Brito and Fishell, 2009). Other studies have instead shown that the majority of dopaminergic OBi, also called small periglomerular cells (PG), are generated during adult life from progenitors located in the VZ-SVZ of the subpallium and cortex (Hack et al., 2005; Kohwi et al., 2005, 2007; De Marchis et al., 2007; Young et al., 2007). During adult neurogenesis, most of the newborn OBi are GCs reinforcing the idea that in general PGCs are born earlier that GCs.

Although OBi are generated in specific neurogenic regions, such as the dLGE and prospective OB, adult neurogenesis occurs in different neurogenic niches. Progenitors located in the VZ and SVZ facing the lateral ventricles of the pallium, striatum, and septum can generate different subclasses of OBi (Corbin et al., 2000; Waclaw et al., 2006, 2009; Fogarty et al., 2007; Inoue et al., 2007; Kelsch et al., 2007; Kohwi et al., 2007; Merkle et al., 2007; Young et al., 2007; Fernández et al., 2011; Weinandy et al., 2011; Figure 3B). It should be emphasized that the primary progenitors of OBi during adult neurogenesis are stem cells, called B1 cells, and contain brain astrocyte-like features, as confirmed by their expression of GFAP and GLAST (Doetsch et al., 1997; Ninkovic et al., 2007). B1 cells can be quiescent or turn into an active state, which is characterized by the expression of Nestin and EGFR (Doetsch et al., 1997, 1999; Codega et al., 2014; Mich et al., 2014). When B1 cells become active, they generate transit-amplifying precursors, also called C cells, which produce at the same time, other C cells and/or neuroblasts (type A cells). Neuroblasts generated in each neurogenic niche differ in their expression of distinct molecular markers (Baker et al., 2001; Saino-Saito et al., 2004; Hack et al., 2005; Kohwi et al., 2005).

To reach the OB, neuroblasts migrate long distances, tangentially and parallel to the cerebral ventricles; this peculiar migratory behavior is known as the Rostral Migratory Stream (RMS) (Altman and Das, 1969; Kishi, 1987; Doetsch and Alvarez-Buylla, 1996; Lois et al., 1996; Figure 3C). While they are migrating, neuroblasts can divide one, probably two more times (Ponti et al., 2013). In the RMS, neuroblasts elongate their cellular aggregates and establish homophilic interactions helping thus other neuroblasts to migrate. This type of behavior called chain migration, is favored by GFAP+ astrocytes covering the neuroblast chains and thus forming a glial tube that neuroblasts use as a scaffold to migrate (reviewed by Gengatharan et al., 2016). The proper formation of glial tubes and the contact between neuroblast and astrocytes with blood vessel are essential for neuroblast migration during adult stages (Soria et al., 2004; Belvindrah et al., 2007; Snapyan et al., 2009; Whitman et al., 2009; Eom et al., 2010; Kaneko et al., 2010). This differs however from embryonic and early postnatal stages, where neuroblasts move without the help of glial tubes or vascularized cells, and indicates that mechanisms involved in regulating migration within the RMS are different depending on the time it occurs (Sun et al., 2010). Another factor that seems to be determinant in neuroblast migration is the neural cell adhesion molecule (PSA-NCAM), essential for the maintenance of neuroblasts in chains (Tomasiewicz et al., 1993; Cremer et al., 1994; Ono et al., 1994; Hu et al., 1996; Chazal et al., 2000; Battista and Rutishauser, 2010). Other cell-cell adhesion and extracellular matrix (ECM) proteins are also implicated in neuroblast chain migration along the RMS. For instance, integrins expressed by neuroblasts guide their migration by interacting with laminins at the ECM. Neuroblasts also avail of chemomolecules expressed by their final target (the OB) and locally within the RMS. The presence of netrin-1 in the OB will assign the migratory direction of neuroblasts expressing the netrin receptor DCC (Murase and Horwitz, 2002). On the contrary, Slit proteins in the septum will act as chemorepulsive for neuroblast expressing Robo receptors (Nguyen-Ba-Charvet, 2004).

Once neuroblasts reach the OB, they migrate radially and integrate into the different OB layers where they will differentiate into interneurons (Figure 3C). The glycoprotein RLN plays a double key role in OBi migration during the last steps of their long journey toward the OB. On the one hand, RLN is primordial for the correct organization of OB layers (Ogawa et al., 1995), and on the other hand, RLN secretion by the mitral and tufted cells is crucial for the radial migration of neuroblasts within the OB (Hack et al., 2002). Other factors implicated in the detachment of neuroblasts from the RMS and in driving them to their correct OB location by radial migration are tenascin-R (Saghatelyan et al., 2004) and prokineticin-2 (Ng et al., 2005).

The RMS is a special migratory path for several reasons. First, because it occurs during adult stages in which the environment is normally not very suitable for neural migration. Second, because migrating cells are proliferative and still able to divide one or two more times while migrating. Another important aspect to mention is that the RMS is one of the longest migratory paths in the CNS, and neuroblasts migrate in chains with a considerable speed when compared to other migratory streams (Wichterle et al., 1997; Nam et al., 2007). However, there are still several open questions in the field, such as whether newborn OBi are involved in olfactory information processing or behavior, and how they become integrated into a mature system without disassemble it (Lledo and Valley, 2016).

## Glutamatergic neuron migration in the telencephalon

Glutamatergic neurons in the telencephalon are generated in the dorsal telencephalon (dTel) or pallium. The pallium is subdivided into medial, dorsal, lateral and ventral pallium (MP, DP, LP, and VP respectively; Puelles et al., 1999, 2000, 2013; Medina et al., 2004). The DP gives rise to the neocortex, while the MP generates the hippocampal formation. The VP and LP pallia also generate neurons that migrate to the claustrum, piriform and entorhinal cortex, as well as to the amygdalar complex (Puelles, 2014; Puelles et al., 2016a). The majority of glutamatergic neurons generated in the VZ and SVZ of the embryonic pallium migrate radially to reach their correct laminar positions. This general mechanism applies to pyramidal neurons in the cerebral cortex, hippocampus and cortical amygdala, as well as to mitral and tufted cells in the OB. All these pallial structures are organized into layers composed of different neurons. The proper radial migration of glutamatergic neurons and therefore, the organization of the cortical layers depends on secreted extracellular molecules, such as RLN (D'Arcangelo et al., 1995; de Rouvroit and Goffinet, 2001; Rice and Curran, 2001), and cell adhesion molecules, such as integrins (Edmondson et al., 1988; Stitt and Hatten, 1990; Fishell and Hatten, 1991; Anton et al., 1997; Adams et al., 2002; Franco et al., 2011; Gil-Sanz et al., 2013).

There are however other glutamatergic populations that migrate tangentially to reach their target structures, such as (1) transitory glutamatergic neurons generated from Dbx1-expressing progenitors at the VP that migrate tangentially to the CP (Teissier et al., 2010, 2012); (2) subplate neurons generated from the rostro-medial telencephalic wall (RMTW) that also migrate caudally (García-Moreno et al., 2008; Pedraza et al., 2014); (3) granule cell precursors that will form the dentate gyrus of the hippocampus (Altman and Bayer, 1990; Nakahira and Yuasa, 2005; Li et al., 2009; Li and Pleasure, 2014; Seki et al., 2014) and (4) glutamatergic neurons generated in the septum that migrate tangentially and caudally through the olfactory tuberculum, olfactory cortex and POA (García-Moreno et al., 2008; Ceci et al., 2012).

### The dorsal pallium (DP) not only generates neocortical pyramidal neurons

The DP is the subdivision of the pallium that will give rise to the future neocortex. The neurons generated from the DP migrate radially in an inside-outside gradient to form the 6-layer neocortical structure. There are different molecules implicated in each step during radial migration of these neurons. The cyclin-dependent kinase 5 (cdK5) and its activators p35 and p39, for example, play key roles in radial-glia guided migration, mainly during nucleokinesis. In *Cdk5* mutant mice, neocortical cells from layers 4 to 2 fail to reach their correct positions, resulting in an inverted cortical layer organization (Ohshima et al., 1996; Gilmore et al., 1998).

**Figure 4:**
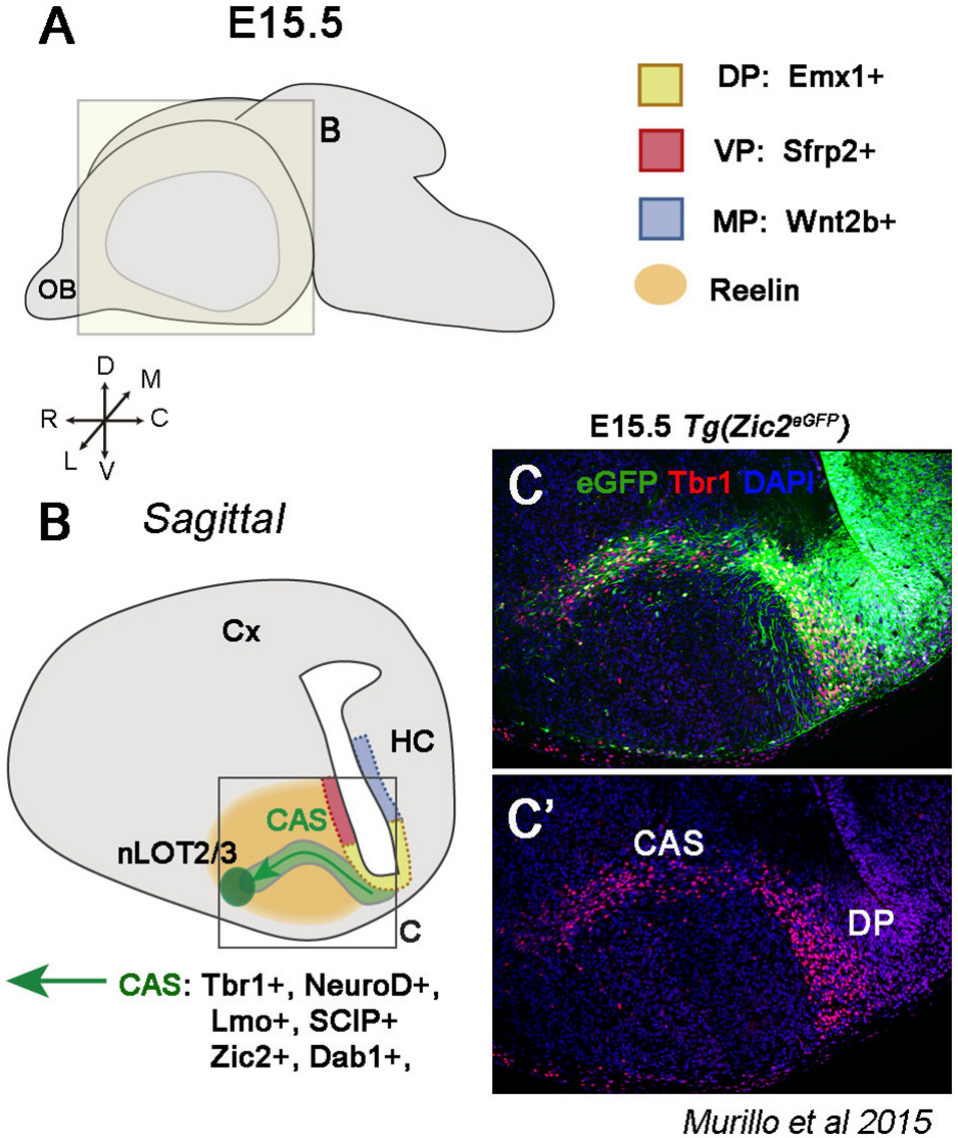
The dorsal pallium (DP) generates glutamatergic nLOT2/3 neurons. **(A)** Schematic of a whole embryonic brain at E15.5 showing the plane for illustrations in **(B,C)**. **(B)** Sagittal section representing the nLOT2/3 cells migration through the caudal amygdaloid stream (CAS). nLOT2/3 cells are generated from the DP characterized by Emx1 expression in the VZ (yellow region). The VZ of the VP (red region and Sfrp2+) and the VZ of the MP (blue region and Wnt2b+) are adjacent territories that do not generate nLOT2/3 neurons. While they are migrating across the CAS, nLOT2/3 cells express NeuroD, Tbr1, Zic2, and Dab1. The glycoprotein reelin (RLN) is expressed around the CAS (orange region) and plays a key role in the guidance of nLOT2/3 cell migration. **(C)** Imunostaining of a region boxed in B illustrating the expression of Zic2 (in green) and Tbr1 (in red) in nLOT2/3 neurons along the CAS.

The DP also generates glutamatergic neurons that will reach the amygdala. For example, the nucleus of the lateral olfactory tract (nLOT) is a 3-layer amygdalar structure originating from different regions. Electroporation experiments have shown that layers 2/3 cells of the LOT nucleus (nLOT2/3) are generated from the most caudal pole of the DP (Remedios et al., 2007). This territory expresses the TF Emx1 and is negative for Sfrp2 and Wnt2b, markers of the VP and MP, respectively, excluding these territories as the possible origin of nLOT2/3 cells (Figures 4A,B). However, nLOT2/3 neurons fail to be generated in mutant mice in which the DP is highly affected, providing thus strong support of a neocortical origin of this population (Hevner et al., 2001; Shinozaki et al., 2002; Remedios et al., 2007). Furthermore, nLOT2/3 neurons are characterized by the expression of characteristic pallial markers, such as Tbr1 (Puelles et al., 2000; Medina et al., 2004; Remedios et al., 2004, Figure 4C), NeuroD (D1, D2, D6), Neurogenin2 (Neurog2), Lmo (1,2,3,4), and SCIP (Remedios et al., 2004, 2007). These neurons are normally generated around E11.5-12.5 (Mcconnell and Angevine, 1983; Remedios et al., 2007; Soma et al., 2009) and then migrate rostrally *via* the Caudal Amygdaloid Stream (CAS) to settle into layers 2/3 of the nLOT (green arrow Figure 4B). RLN controls nLOT2/3 migration similarly to pyramidal cells in the CP; however, in this case, RLN is expressed around the CAS and not within the cell stream itself, forming in this way a RLN-negative corridor. To respond to RLN, nLOT2/3 cells express the cytosolic component Disabled homolog 1 (Dab1), and in *reeler* mice (mice that are *null* for RLN or RLN cannot be secreted), the CAS fails to form the characteristic V shape, has an aberrant position and nLOT2/3 cells are located more dorsally than expected (Remedios et al., 2007).

Notably, CAS neurons seem to migrate in two distinct phases, tangentially and radially. In a first phase, neurons migrate parallel to the VZ and therefore orthogonal to the radial glia. In a later phase, cells turn and locate themselves along the radial glia, forming the characteristic V-shaped path. But do nLOT2/3 cells use radial glia to migrate during the late phase? As previously mentioned, radial glia-dependent migration is controlled by the protein kinase Cdk5. In *Cdk5* mutant mice, CAS neurons stop at the level of the second phase migration, but other amygdaloid nuclei are formed at appropriate locations (Remedios et al., 2007), implying that CAS neurons can migrate parallel to the ventricular surface, but are not able to form the second phase migration. This, together with the requirement of RLN for proper migration, strongly suggests that nLOT2/3 cells use similar migratory mechanisms as other DP derivatives. However, these mechanisms are not required in the migration of other components of the pallial amygdala, supporting the different origin of this population.

Another key factor implicated in the correct migration of nLOT2/3 neurons is a member of the ZIC family, Zic2, which is implicated in axonal guidance of retinal ganglion cells, thalamocortical axons, and midline crossing of dorsal spinal cord neurons (Herrera et al., 2003; García-Frigola et al., 2008; Escalante et al., 2013). A recent study has shown that Zic2 is also required in cell migration in the telencephalon (Murillo et al., 2015). Zic2 is expressed in Cajal-Retzius cells of the cortical surface and in migrating nLOT2/3 cells along the CAS (Murillo et al., 2015) (Figure 4C). CAS cells migrate aberrantly without forming the typical V-shaped stream in *Zic2* hypomorphic mice and occupy a much wider area (Murillo et al., 2015). Therefore, nLOT2/3 cells use several migratory mechanisms before reaching their final destination.

### Tangential migration of cajal-retzius cells (CRCs) along the cortical surface

The migration of CRCs exhibits special characteristics that are worth mentioning. CRCs represent a transitory population playing key roles in the formation of cortical layers and neural circuitries by secreting RLN (D'Arcangelo et al., 1995; Ogawa et al., 1995; Rice and Curran, 2001). For a long time, it was thought that CRCs were generated in the DP, however nowadays there is a real consensus on their origin at the edges of the pallium. The cortical hem, the VP and the pallial septum generate molecular, morphological and electrophysiological different subpopulations of CRCs that will migrate tangentially to be distributed along the entire cortical surface (Yoshida et al., 2006; Tissir et al., 2009; Griveau et al., 2010; Sava et al., 2010; Zimmer et al., 2010; Dixit et al., 2011). This implies that CRCs generated in the pallial septum, next to the anterior neural ridge, will migrate from rostral to caudal, whereas cortical-hem derived CRCs will migrate from caudal to rostral and VP ones from lateral to dorso-medial cortex (Takiguchi-Hayashi et al., 2004; Bielle et al., 2005; García-Moreno et al., 2007; Gu et al., 2009; Griveau et al., 2010; Barber et al., 2015). But unlike other cell populations described in this review, CRCs do not follow signaling cues to any specific direction. Indeed, they are distributed along the cortical surface by contact repulsion mediated by ephrin interactions (Villar-Cerviño et al., 2013). Nevertheless, some environmental factors can influence their migration (Ceci et al., 2010). For example, CXCL12 expressed by meninges control CRC dispersion in the cortical primordium *via* the CXCR4 receptor (Borrell and Marín, 2006; Paredes et al., 2006). On the other hand, Sema3D\PlexinD1 signaling controls the motility of CRCs modulating CXCL12/CXCR4 signaling (Bribián et al., 2014). Radial glia cells in the cortex are also important for their localization in the MZ (Kwon et al., 2011), and intrinsic mechanisms, such as expression of the TFs Ebf2 and Zic2, influence CRC motility, and therefore the final distribution of hem- and septal-derived CRCs (Chiara et al., 2012; Murillo et al., 2015)

Although CRCs migrate randomly along the cortical surface, they maintain a certain gradient distribution. The higher density of septal CRCs is localized mainly in the rostral cortical region, hem-derived CRCs are preferably in medial and caudal cortex, and VP ones are distributed mainly in the lateral cortex (revised in Barber and Pierani, 2016). The gradual distribution of the different CRC populations seems to play a role in cortical arealization. Ablation of one of the these subpopulations or changes in their migration speed partially affects cortical arealization, suggesting that CRCs can act as mobile patterning units during area mapping (Borello and Pierani, 2010; Griveau et al., 2010; Barber et al., 2015; Barber and Pierani, 2016).

Finally, there is also a special CRC subpopulation that migrates from caudal to rostral following a specific migratory path controlled by several chemomolecules. These are the CRCs generated in the thalamic eminences and described below in the next paragraph.

### The lateral thalamic eminence (LTE) generates glutamatergic neurons migrating to the olfactory system and cortex

**Figure 5:**
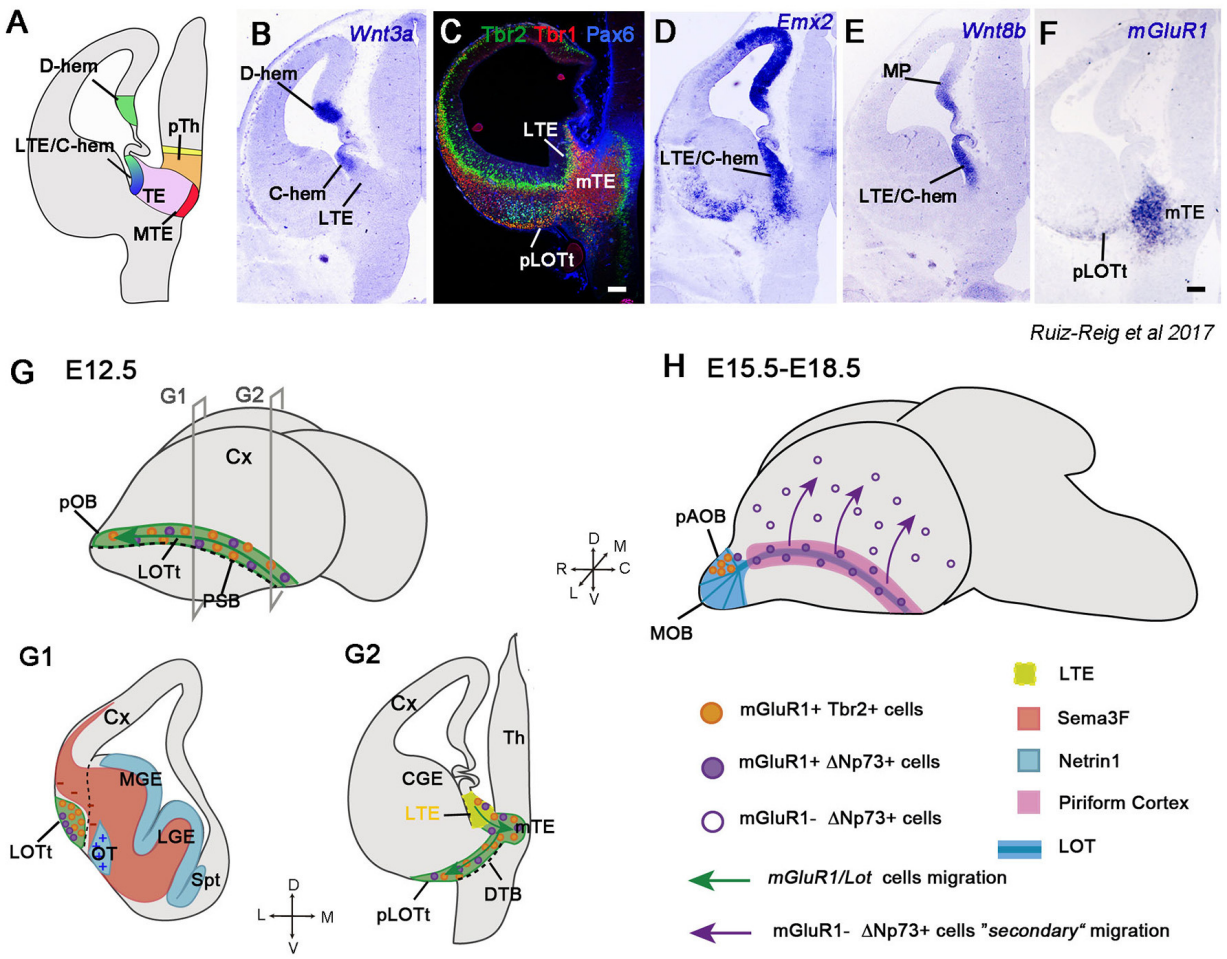
The long route of the *mGluR1/Lot* cells toward the olfactory bulb (OB) and cortex. **(A)** Schematic of a coronal section at E12.5 showing the different structures around the TE. **(B–F)** Coronal sections illustrating the expression of Wnt3a, Tbr2, Tbr1, Pax6, Emx2, Wnt8b, and mGluR1 (Grm1) in the LTE, caudal hem (C-hem) and TE mantle at E12.5. Images taken from Ruiz-Reig et al. (2017). **(G)** Schematic of a whole embryonic brain at E12.5 showing the migratory path taken by the *mGluR1/Lot* cells along the prospective LOT territory (LOTt), and indication of the planes used for G1 and G2 illustrations. The dashed line represents the PSB. **(G1,G2)** *mGluR1/Lot* cells are generated from the LTE (yellow region) and then migrate to the mTE crossing the diencephalon-telencephalon boundary (DTB) to reach the posterior tier of the LOTt. Once they reach the LOTt, *mGluR1/Lot* cells migrate rostrally through this territory along the PSB. The *mGluR1/Lot* cell population is composed mainly of cells expressing mGluR1 and Tbr2 (the prospective pAOB mitral cells, orange dots) and of a small population expressing mGluR1 and ΔNp73 (Cajal-Retzius cells, violet dots). Netrin 1 (represented in light blue color) is expressed in the VZ of the ganglionic eminences and in the olfactory tubercle and acts as an attractant cue for *mGluR1/Lot* cells. The semaphorin Sema3F (represented in orange/red color) is a repulsive cue for migrating *mGluR1/Lot* cells and it helps in confining this population to the LOTt. **(H)** Schematic of a whole embryonic brain between E15.5 and E18.5. At E15.5, the prospective pAOB mitral cells have reached the posterior tier of the AOB and the *lot-*Cajal-Retzius cells are located around the AOB and in the piriform cortex (pink area). A secondary migration of *lot-*Cajal-Retzius occurs when they switch off mGluR1 expression (empty violet dots), and then migrate dorsally from the piriform cortex to the neocortical surface (violet arrows).

The dorsal part of the telencephalon, the pallium, is very expanded in mammals with a highly sophisticated cerebral cortex. The cortical hem, located in the roof of the telencephalon, is continuous with the lateral part of the thalamic eminence (TE) in its most caudal edge (Ruiz-Reig et al., 2017; Figure 5A). Although the TE is located in prosomere 3 of the diencephalon, the lateral subdomain of the TE can be considered a transitory zone between the telencephalon and the diencephalon. Moreover, the TE and in particular the lateral TE (LTE), has pallial characteristics, as confirmed by the expression of the pallial markers Pax6, Emx2, Wnt8b, Ngn2, Tbr2, and Tbr1, and hence, with features more of telencephalic structures than of diencephalic ones (Ruiz-Reig et al., 2017; Figures 5A–E). The TE is a source of cells positive for the metabotropic glutamate receptor mGluR1 destined to the piriform cortex and olfactory bulbs (Huilgol et al., 2013; Ruiz-Reig et al., 2017; Figure 5F). This population can act as guidepost cells for the lateral olfactory tract (LOT) and are therefore called *lot cells* (Sato et al., 1998). The *lot* cells were visualized for the first time using the monoclonal antibody (mAb) lot1 that recognizes the glutamate metabotropic receptor mGluR1 (Sato et al., 1998; Hirata et al., 2012), and for this reason they are also named *mGluR1/Lot* cells. They were believed to originate from the DP (Tomioka et al., 2000; Kawasaki et al., 2006; Ito et al., 2008), but a recent study showed strong evidence that their main origin is the TE (Ruiz-Reig et al., 2017). These cells are generated specifically from the LTE and then migrate ventrally through the diencephalic-telencephalic boundary (DTB) to the posterior part of the prospective LOT territory (pLOTt), the area where the LOT will develop (green region in Figure 5G). Once they reach this region, *mGluR1/Lot* cells migrate rostrally along the external PSB (Figures 5G,G1,G2). These cells represent a heterogeneous neural population composed of precursors of the posterior accessory olfactory bulb (pAOB) mitral cells and of a small portion of ΔNp73+/mGluR1+ cells, most likely CRCs (Huilgol et al., 2013; de Frutos et al., 2016; Ruiz-Reig et al., 2017). Besides mGluR1, the two populations share the expression of the glycoprotein RLN and the TFs Tbr1 and Lhx5 (Ruiz-Reig et al., 2017).

From around E13.5, precursors of the pAOB cells continue to migrate rostrally to populate the pAOB, where they will integrate as mitral cells. During this migration, they express some general mitral cell markers, such as Tbr2, Tbr1, and RLN, and specific markers of the pAOB, such as Lhx5, AP-2α and COUP-TFI. In fact, AP-2α and COUP-TFI expressions define two waves of migrating neurons. While the first neurons reach the pAOB (E13.5) and are characterized by AP-2α expression, the second migration of COUP-TFI-positive/AP-2α-negative cells will colonize the pAOB two days later (E15.5) (Ruiz-Reig et al., 2017). Nevertheless, a small fraction of mGluR1+ cells situated in the piriform cortex at E12.5 express the TF ΔNp73, but is negative for Tbr2. This discrete population (around 15–18% of the mGluR1+ population located in the LOT region at E12.5) is likely to represent CRCs migrating through the piriform cortex. The mGluR1+p73+ cells in the LOT territory are also generated from the LTE and migrate through the LOTt to disperse between the MZ of the piriform cortex and the external region of the AOB (Ruiz-Reig et al., 2017). A recent work shows a secondary tangential migration of this population toward the neocortex at around E14.5-E15.5, at a time when they downregulate mGluR1 expression (violet arrows in Figure 5H). This late migration supplies a subpopulation of CRCs to the cortex essential for the correct wiring of neocortical circuitry (de Frutos et al., 2016).

The exact location of the *mGluR1/Lot* cells is controlled during development by multiple guidance molecules. They express the receptor Nrp2, whereas Sema3F is present in the mantle zone of the GE and avoids *mGluR1/Lot* cells to enter these territories, thus confining them to the LOTt (red region in Figure 5G1). In *Nrp2*- or *Sema3F*-deficient mice (*Nrp2*^−/−^, *Sema3F*^−/−^), *mGluR1/Lot* cells are more spread and abnormally invade the GE; however, even if they are in aberrant positions, the formation of the LOT seems to be normal (Ito et al., 2008). Netrin1-DCC signaling is also implicated in the correct positioning of the *mGluR1/Lot* population. Netrin1 is distributed in the VZ of the GE and in the olfactory tubercle (blue region in Figure 5G1), whereas its receptor DCC is expressed in *mGluR1/Lot* cells. However, in *Netrin1*- and *DCC*-deficient mice (*Netrin*^−/−^, *DCC*^−/−^), the location of *mGluR1/Lot* cells is only weakly affected in the LOTt, and LOT axons have modest deficits in their trajectories (Kawasaki et al., 2006).

Posterior AOB mitral cells and *mGluR1*^+^ CRCs are generated from the LTE and use the same migratory path to reach the piriform cortex and the AOB. It is interesting to note that the LTE, similarly to other CRC origins, is situated at the edge of the pallium and is proposed to act as a signaling center for forebrain patterning (Adutwum-Ofosu et al., 2016). Therefore, the TE can be considered part of the hem system together with the VP, the cortical hem and the septum (Roy et al., 2014).

## Conclusions and perspectives

In this review, we have discussed different populations that migrate long distances during mouse brain development before being incorporated into distinct neuronal circuitries. However, an obvious question that comes up is: why are these neurons not generated locally? The answer most probably lies within the complexity of the nervous system. Different regions of the telencephalon are specialized in generating distinct classes of neurons, and conversely, the nervous system needs several types of cells to properly work; thus, neurons will migrate short or long distances depending on where they are needed. The most representative case comes from the OB, which is an evagination of the rostralmost part of the cortex. The OB receives at least three neural populations not generated locally: CRCs, OBi, and pAOB mitral cells. OBi are generated in the subpallium far away from the OB, and therefore they must migrate tangentially and rostrally to reach the OB through the RMS.

But why are the mitral cells of the posterior tier of the AOB generated in the LTE? One hypothesis could be that this population retains specific features essential for the information processing implicated in aggressive/defensive behaviors, whereas mitral cells located in the anterior AOB are required for processing information related to mating behaviors (Halpern and Martínez-Marcos, 2003). Another feasible hypothesis would lie in the fact that both, the TE and the AOB, are very evolutionary conserved structures. For instance, the TE is already present in non-amniotic vertebrates (fish and frog), whereas other structures more related to the neocortex, such as the cortical hem, is absent (Roy et al., 2014). In mammals, the pallium is the most complex and, in terms of evolution, the most recent structure, which hugely increases in size by anatomically keeping the TE away from the OB (Medina and Abellán, 2009). However, in other vertebrates such as the lizard, the OB is very large and the TE seems to form a continuum with the pallial septum making the migration from the TE to the AOB more feasible (Desfilis et al., 2017). Indeed, the migratory pathway from the TE toward the AOB is conserved in non-amniotic vertebrates, as observed in the frog *Xenopus laevis* (Huilgol et al., 2013).

The amygdaloid complex is also an evolutionary conserved structure related to the olfactory system (Medina et al., 2011). We have discussed how different GABAergic populations, generated in the ventral MGE and POA, migrate caudally to colonize the postero-medial amygdala besides other caudal structures. On the other hand, glutamatergic neurons generated from the DP migrate in opposite directions to integrate into nLOT2/3 neurons. The medial amygdala receives information from the AOB and is thus considered to be part of the vomeronasal amygdala, whereas nLOT cells receive inputs from the main OB.

Finally, there are other neuronal populations with extra-telencephalic origins that cross the telencephalon along their migratory trajectories. For instance, the Gonadotropin-releasing hormone (GnRH) neurons are generated in the olfactory placode/vomeronasal organ and then migrate along the caudal branch of the vomeronasal nerve to reach the hypothalamus (Schwanzel-Fukada and Pfaff, 1989; Wray et al., 1989). On the contrary, OTP-expressing neurons generated in the lateral hypothalamus migrate rostrally and then caudally to finally settle in the medial and postero-medial cortical amygdala (García-Moreno et al., 2010).

All these migratory paths have in common that they need to travel long distances during development or during adult life, as is the case of OBi. Longer distances in cell migration imply that neurons in these streams will cross several environments and, therefore will need to continuously change their strategies in order to reach their appropriate targets. We just start to understand what are the factors implicated in each step of their migratory routes. Mutations of one of these cues could lead to defective assembly of neural circuitries, and thus be involved in neurodevelopmental diseases and/or psychiatric disorders, whose symptoms usually appears at later stages. For instance, recent studies have shown that cIN dysfunction is directly related to psychiatric disorders and depression (Marín, 2012; Fee et al., 2017). Defects in cIN generation and migration will lead to altered number, ratio or integration of these neurons in the cortical network producing thus an imbalance between excitatory and inhibitory activity. For example, COUP-TFI and COUP-TFII are implicated in the generation of different cINs by promoting either SST identity for COUP-TFII, or specifying VIP+ and CR+ cINs (i.e., CGE identity) for COUP-TFI (Lodato et al., 2011; Hu et al., 2017). In addition, both TFs control the tangential migration of ventral telencephalic cells (Tripodi et al., 2004) and guide CGE neurons to their different migratory streams (Touzot et al., 2016). COUP-TFI and COUP-TFII expression profiles in interneurons are comparable between fetal human brains and embryonic mice (Alzu'bi et al., 2017a,b), making these nuclear receptors very good candidates directly implicated in the integration of inhibitory interneurons in the human cerebral network.

An important psychiatric condition, which has a high prevalence in human patients, is the Autism spectrum disorder (ASD). Individuals with ASD present neuroanatomical alterations due to the vast heterogeneity of developmental defects; one of them is the diminution of PV+ interneurons in the prefrontal cortex (Ariza et al., 2016; Hashemi et al., 2017). These interneurons, represented by chandelier and basket cells, are generated in the MGE and then migrate to the neocortex by tangential migration. Decreased number of PV+ interneurons produces an imbalance between excitation and inhibition in the cortical network, responsible for some of the ASD symptoms (revised by Rubenstein and Merzenich, 2003; Varghese et al., 2017). Interestingly, another structure affected in ASD patients is the amygdala, which has fewer neurons compared to normal individuals (Schumann and Amaral, 2006) and could explain some of the cognitive and social deficits characterized by ASD. In particular, the medial amygdala plays a fundamental role in social-aggressive behaviors. This part of the amygdala is composed by GABAergic and glutamatergic neurons generated in several regions during development, such as the POA, the cvMGE and the lateral hypothalamus, before converging into the different MeA subnuclei *via* migration. Similarly to the neocortex, alterations in the generation, migration and integration of one population could lead to imbalanced excitatory and inhibitory activity in the amygdala circuitries, causing potential social behavior deficits, one of the major symptoms observed in patients affected with ASD.

## Author contributions

All authors listed have made a substantial, direct and intellectual contribution to the work, and approved it for publication.

### Conflict of interest statement

The authors declare that the research was conducted in the absence of any commercial or financial relationships that could be construed as a potential conflict of interest.

## Abbreviations

AEP: antero endopeduncular area
AM: amygdala
C: caudal
CAS: caudal amygdaloid stream
CGE: caudal ganglionic eminence
cIN: cortical interneurons
CMS: caudal migratory stream
CNS: central nervous system
CP: cortical plate
CRCs: Cajal-Retzius cells
cvMGE: caudo-ventral MGE
Cx: cortex
D: dorsal
Dg: Diagonal area
dLGE: dorsal LGE
dMGE: dorsal MGE
DP: dorsal pallium
DTB: diencephalon-telencephalon boundary
dTel: dorsal telencephalon
GCs: Granule cells
HC: hippocampus
L: lateral
LGE: lateral ganglionic eminence
LMS: lateral migratory stream
LOT: lateral olfactory tract
LOTt: LOT territory
LP: lateral pallium
LTE: lateral thalamic eminence
LV: lateral ventricle
M: medial
MeA: Medial nucleus of the amygdala
MePD: posterodorsal medial amygdala
MePV: posteroventral medial amygdala
MGE: medial ganglionic eminence
MMS: medial migratory steam
MOB: main olfactory bulb
MP: medial pallium
mPOA1: POA1 mantle
MTE: medial thalamic eminences
mTE: thalamic eminence mantle
MZ: marginal zone
nLOT2/3: layer 2 of the nucleus of the LOT
OB: olfactory bulb
OBi: olfactory bulb interneurons
OT: Olfactory tubercle
pAOB: posterior accessory olfactory bulb
PAS: preoptic amygdala stream
PGCs: periglomerular cells
pLOTt: posterior LOT territory
POA: preoptic area
pOB: prospective olfactory bulb
pPOA2: proliferative area of POA2 region
PSB: pallial-subpallial boundary
R: rostral
RGCs: radial glia cells
RMS: rostral migratory stream
Spt: septum
Str: striatum
SvSpM: subventricular subpallial migratory stream
SVZ: subventricular zone
TE: thalamic eminence
TF: transcription factor
Th: thalamus
V: ventral
vLGE: ventral LGE
vMGE: ventral MGE
VP: ventral pallium
vTel: ventral telencephalon
VZ: ventricular zone.
